# Spinoculation and retronectin highly enhance the gene transduction efficiency of Mucin-1-specific chimeric antigen receptor (CAR) in human primary T cells

**DOI:** 10.1186/s12860-021-00397-z

**Published:** 2021-11-23

**Authors:** Alireza Rajabzadeh, Amir Ali Hamidieh, Fatemeh Rahbarizadeh

**Affiliations:** 1 Department of Applied Cell Sciences, Faculty of Medicine, Kashan University of Medical Sciences, Kashan, Iran; 2 Pediatric Cell and Gene Therapy Research Center, Gene, Cell and Tissue Research Institute, Tehran University of Medical Sciences, Tehran, Iran; 3 Department of Medical Biotechnology, Faculty of Medical Sciences, Tarbiat Modares University, Tehran, Iran; 4 Research and Development Center of Biotechnology, Tarbiat Modares University, Tehran, Iran

**Keywords:** Chimeric antigen receptor, Immunotherapy, Polybrene, Retronectin, T cell

## Abstract

**Background:**

Producing an appropriate number of engineered cells is considered as one of the influential factors in the successful treatments with chimeric antigen receptor (CAR) T cells. To this aim, the transduction rate of the viral vectors can play a significant role. In addition, improving transduction rates can affect the success rate of this treatment due to hard-transduced T lymphocytes.

**Results:**

In this study, activated T cells were transduced using different transduction methods such as spinoculation, retronectin, polybrene, spinoculation + retronectin, and spinoculation + polybrene after selecting the most efficient transfection method to produce recombinant viral particles containing MUC1 CAR. PEI and lipofectamine with the amount of 73.72 and 72.53%, respectively, showed the highest transfection rates with respect to calcium phosphate (14.13%) for producing lentiviral particles. However, the cytotoxicity of transfection methods was not significantly different. Based on the results, spinoculation + retronectin leads to the highest transduction rates of T cells (63.19 ± 4.45%) relative to spinoculation + polybrene (34.6 ± 4.44%), polybrene (10.23 ± 0.79%), retronectin (10.37 ± 1.85%), and spinoculation (21.11 ± 1.55%). Further, the polybrene (40.02%) and spinoculation + polybrene (48.83% ± 4.83) increased cytotoxicity significantly compared to other groups.

**Conclusion:**

Improving transduction conditions such as using spinoculation with retronectin can ameliorate the production of CAR-T cells by increasing the rate of transduction, as well as the success rate of treatment.

**Supplementary Information:**

The online version contains supplementary material available at 10.1186/s12860-021-00397-z.

## Background

During the recent years, cancer immunotherapy, especially hematologic malignancies, has created promising clinical outcomes. The use of genetically modified T lymphocytes targeting CD19 leads to remission in 70–90% of children with B-cell acute lymphoblastic leukemia (B-ALL) [1, 2]. These redirected T cells express a synthetic receptor called the CAR which recognizes the CD19. CAR structure comprises an antigen-recognizing domain (derived from single-chain variable fragments of antibodies), a hinge sequence, and intracellular signaling domains.

Mucin-1 (MUC1) is considered as a large membrane-bound glycoprotein which is aberrantly overexpressed in most human breast cancers and other malignancies such as pancreatic cancer, lung carcinoma, multiple myeloma, ovarian cancer, etc. [3]. MUC1 has been attractive for immunotherapies since the overexpression of this antigen is related to increased tumor aggressiveness and poor prognosis tumors. Redirecting T lymphocytes against MUC1-positive breast cancer cells by CAR technology was first developed and characterized by Scott Wilkie et al. and indicated that the MUC1-targeting CAR T cells efficiently eliminate breast tumors [4]. Most recently, a novel CAR based on a humanized antibody (5E5), which specifically recognized Tn (GalNAca1-O-Ser/Thr) glycoform of MUC1 was developed. The results demonstrated that the 5E5-based CAR T cells significantly targeted multiple types of cancer cells expressing the Tn-MUC1, including breast cancer cells, leukemia cells, and pancreatic cancer cells, [5].

Gene engineering of primary T cells to express CAR requires the delivery of CAR construct gene into T cells. Integrative viral gene delivery methods are superb due to stable transgene expression and high transduction efficacy of the possibility of large-scale production using packaging cell lines although CAR gene transfer using transient methods such as mRNA electroporation or adenovirus-associated vector delivery system is safer [6].

On the other hand, developing the third generation of lentiviral vectors named SIN (self-inactivating) vectors has largely alleviated safety concerns, which has been approved for human use in some cases such as the Kymriah [7].

CAR-T cells are now made mainly through viral vectors, especially lentiviruses. The ability to infect non-proliferative cells and difficulty of infect cells is the advantage of lentiviral vectors over gamma retroviral vectors Delivery of the CAR gene into the T cell by lentiviral vectors involves the production of pseudoviruses containing the CAR transgene. The helper plasmids such as gag, pol, rev, and VSV-G genes, and the transgenic plasmid are transiently transfected into packaging cells such as HEK-293 T to produce the recombinant virus. Recombinant pseudoviruses are packaged and made in the cytoplasm of cells and released around the cell after destroying the cell membrane. Different methods such as calcium phosphate, cationic lipids, or polyethyleneimine are used for transfecting plasmids. Meanwhile, the high cost or complexity of these methods prevents from their use in a large-scale system although using cationic lipids and calcium-phosphate leads to high transfection rates in lab-scale setups [7].

Currently, clinical settings of CAR T technology use the third generation of lentiviral vectors for gene engineering of T cells. One of the significant problems in producing CAR T cells is that lentiviral vectors cannot infect quiescence and naïve T cells effectively and integrate the CAR gene into the genome. Therefore, it is necessary to activate the pre-transduction of naïve T cells. In addition, high multiplicities of infection (MOI) of the virus are used by standard protocols to overcome the limitations of hard-transduced cells like primary T cells. The use of high concentrations of the virus (viral MOI) results in increasing the time and cost of producing CAR T cells, which can affect the success of treatment [8].

Improving the transduction conditions can ameliorate the efficiency of engineered T cell production leading to the success of the CAR T cell-based therapy since the low rate of T cell transduction is considered as one of the main problems in CAR T cell manufacturing. Hence, T cells were exposed to different transduction conditions in order to improve the transduction rate. In this study, lentiviral transduction was used for the engineering of human T lymphocytess due to efficiency and safety. This study aims to describe the details of the production of lentivirus particles containing CAR MUC1 transgene and the conditions for the high efficiency of human T cell transduction and compare different transduction methods.

## Results

### Transfection of HEK-293 T cells

The previously optimized PEI transfection [9] was compared with the main conventional transfection methods to determine a higher efficacy method of viral production. For this purpose, a total amount of pLJM1-EGFP: pRSV-Rev:pMDLg/pRRE:pMD2.G complex plasmids at 1μg/well was tested in 24 well plates.

**Fig. 1:**
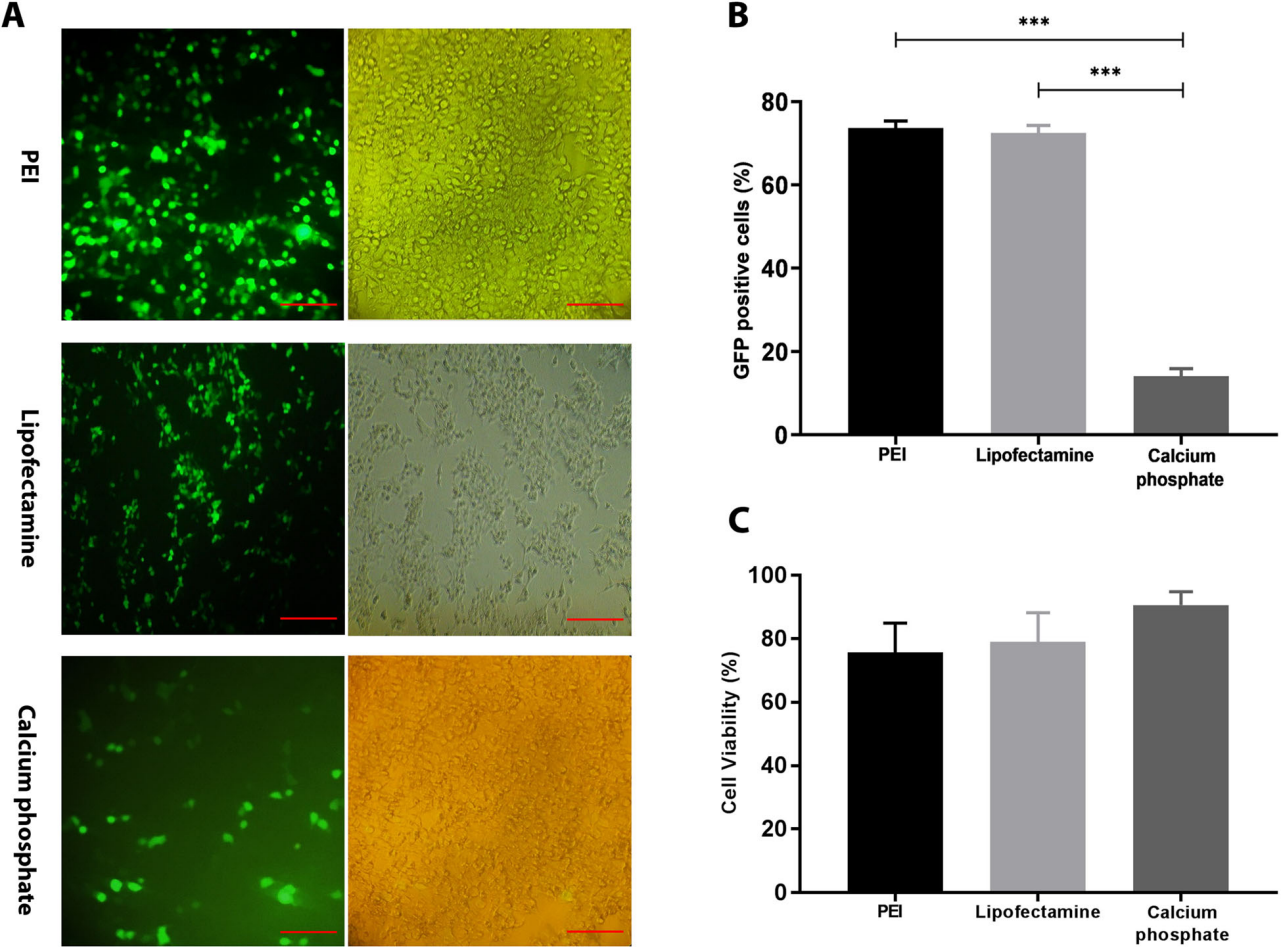
The comparison of transfection efficiency between PEI, lipofection and calcium phosphate methods. **A**) Co-transfection of pLJM1-EGFP:pRSV-Rev:pMDLg/pRRE:pMD2.G plasmids into HEK-293 T cells. Fluorescence microscopy of GFP indicates higher expression levels of GFP in PEI and lypofectamine transfection methods than calcium phosphate method. **B**) Transfection rate of mentioned methods. **C**) The effect of transfection methods on viability of HEK-293 T cell. The scale bar represents 100 μm

Fluorescence microscopic examination of transfected HEK- 293 T cells showed most PEI-based (73.72%) and lipofectamine-based (72.53%) transfectant cells expressing GFP. In contrast, the cells transfected calcium phosphate had low levels of GFP expression (14.13%). (Fig. 1A and B).

Trypan blue dye exclusion was used for evaluating cell viability. The value was not significant although the toxicity of the calcium phosphate method (90.59 ± 4.29% viability) was less than that of transfection methods based on PEI (75.74 ± 9.24% viability) and lipofectamine (79.08 ± 9.15% viability) (Fig. 1C).

### Packaging and producing recombinant lentivirus particles containing MUC1 CAR

Transfer plasmid (pLJM1-CAR MUC1) and packaging/envelope plasmids were transiently transfected to packaging cells (HEK-293 T) using the polyethyleneimine. Then, the media containing recombinant lentiviruses were harvested after 72 h. In addition, lentiviral titer was determined by transducing 8 × 10^4^ HEK- 293 T cells using 20 μL of concentrated virus particles. To this aim, the copy number of the puromycin resistance gene was measured by the absolute quantitative PCR technique. The titer of recombinant CAR MUC1 lentiviral vector in transforming unit per mL was 1.12 × 10^8^ TU/mL from real-time PCR results (Supplementary data).

### Transduction of T lymphocytes

**Fig. 2:**
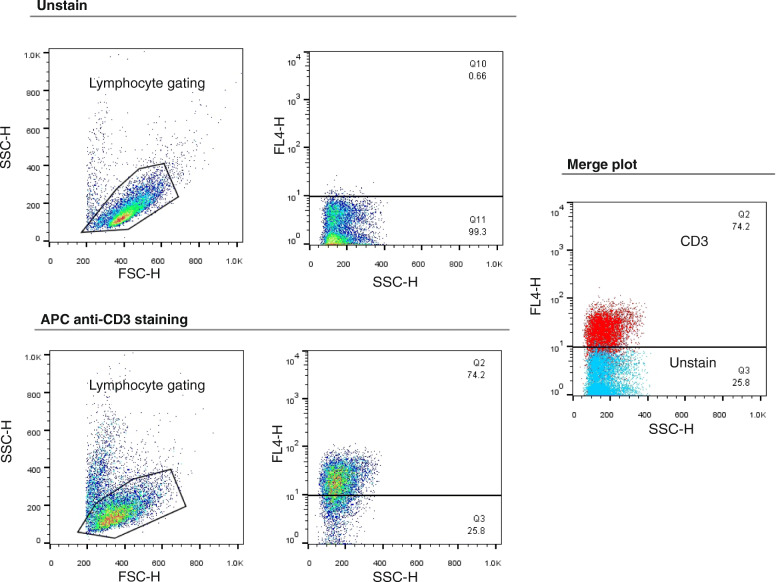
T cell population after stimulation by Dynabeads™ T-Activator CD3/CD28

**Fig. 3:**
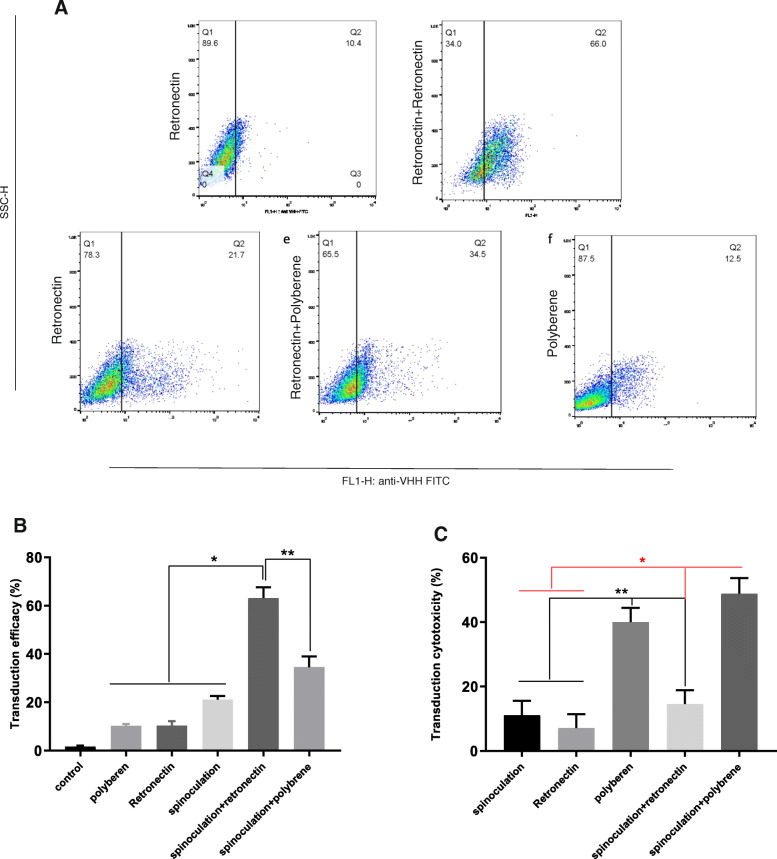
Expression rate of CAR MUC1 on T cells and transduction efficacy. **A**) CAR MUC1 expression in various transduction methods using immunostaining of FITC-conjugated anti-VHH. **B**) Transduction rate of recombinant lentiviral vectors containing CAR MUC1 using various methods. **C**) Cytotoxicity rate of various transduction methods on T cells

As shown in Fig. 2, the stimulation of PBMCs using DynabeadsTM T-Activator CD3/CD28 led to 74.9% CD3 positive cell populations (Fig. 2). Here, five distinct methods were used for transducing the stimulated T lymphocytes. Finally, the surface expression of the chimeric receptor (CAR MUC1) was measured by flow cytometry. Based on the flow cytometric evaluations, the activated T cells transduced by spinoculation + retronectin showed that 63.19 ± 4.45% of the cells expressed the CAR MUC1. However, 34.6 ± 4.44% of cells exposed to viral particles using spinoculation + polybrene indicated the expression of this chimeric receptor. CAR expression reduced significantly in the groups used polybrene (10.23 ± 0.79%), retronectin (10.37 ± 1.85%), or spinoculation (21.11 ± 1.55%) (Figs. 3A and B). An increase in the surface expression of the receptor was significant in the spinoculation + retronectin group compared to the other groups. However, an increase in expression in the spinoculation + polybrene was insignificant compared to the polybrene, retronectin, and spinoculation groups. The results indicated that viral infection and transduction rates were more efficient and effective for T cells in spinoculation with retronectin compared to other groups.

### Cytotoxicity of transduction methods

Cell viability was evaluated for determining the toxicity of different transduction methods on human T cells. In addition, the cytotoxicity rate of polybrene (40.02%) and spinoculation + polybrene (48.83% ± 4.83) increased significantly compared to the other groups. More cytotoxicity was observed in spinoculation (11.14% ± 5.44) and spinoculation + retronectin (14.6% ± 4.30) compared to retronectin (7.11% ± 4.76), although it was insignificant. Based on the results, polybrene had a greater toxicity effect on human T cells during 72 h compared to retronectin. However, spinoculation had no effect on toxicity (Fig. 3C).

## Discussion

In general, viral lentiviral vectors are produced in delayed titration and transient titration methods. The first method requires developing stable packaging cell lines, while viral agents are made transiently in the host cells in the second approach. There are some limitations such as the cytotoxic effects of vector components (gag, pol, rev, and VSV-G), relatively low efficiencies, and an extended selection period characterization complicate their use although stable cell line packaging methods are reproducible and may be cost-effective. In addition, each desired viral vector should develop a new stable cell line with different conditions. In contrast, the transient transfection method of lentiviral plasmids is faster and has higher vector titers [10]. Therefore, in this study, third-generation lentiviral vectors containing CAR transgene were generated using the transient method. In the transient titration method, the transfection rate of transgenic and helper plasmids to packaging cells are vital in virus production. The choice of transfection method can effectively titrate the produced viral particles due to the type of vector and cell line packaging. For this purpose, PEI, calcium-phosphate, and lipofectamine methods were compared for the transient transfer of viral plasmids to packaging cells (HEK-293 T).

The PEI transfection method used in this study was already optimized in the previous study [9]. The results showed that the transfection rates are identical in PEI and lipofectamine methods under similar conditions. However, the calcium phosphate method indicated a significant decrease compared to the other two groups. PEI is inexpensive and more cost-effective although both PEI and lipofectamine methods showed high transfection rates for the pLJM1-EGFP. In addition, PEI is preferred for clinical purposes since methods such as lipofectamine are either challenging to scale up or expensive [7]. Further, Sou et al. (2013) showed that the transfection rate in the PEI method was higher than lipofectamine in different conditions (48 h after transfusion) [11]. Pirona et al. (2020) reported a higher rate of lipofectamine transfection than PEI in adherent cells [12], the difference of which may be related to the differences in the used cell or vector. In the present study, the PEI transfection rate increased significantly compared to the CaPO4 transfection. Furthermore, the PEI-based transfection method is more straightforward than the CaPO4 method and is pH-independent [10]. As a result, PEI was used to produce recombinant lentiviral vectors containing MUC1 CAR transgene and evaluate transduction methods.

The transduction process begins with the attachment of viral particles to the surface of the target cell. This association is formed through specific bindings between virus envelope proteins and their receptors at the cell surface. However, several studies have shown that the initial binding of the virus to the cell membrane is dependent on the binding of the envelope protein to its specific receptor. Non-receptor binding is involved in the formation of this binding [13]. The initial virus-cell binding is affected by the electrostatic repulsion forces between the cell surface and the virus envelop proteins, affecting the efficiency of this junction and, consequently, the transduction efficiency [11]. Recently, polypeptides have been developed for increasing the binding efficiency between viral particles and cells. Retronectin is one of these polypeptides provided by Takara biotech. Since most polycations are cytotoxic, retronectin was reported to be more effective for transducing sensitive and hard-to-transduce cells such as lymphocytes and hematopoietic stem cells [14]. Accordingly, T cell transduction was divided into five groups, and the results were compared with each other. The highest transduction rate of human activated T cells was observed in the spinoculation + retronectin group. Considering that the transfection rate was low in the groups without spinoculation and no significant difference was observed, the spinoculation process could play an essential role in the efficiency of virus binding to T lymphocytes, as well as in the transduction rate.

So far, several studies have been directed to clear these mechanisms. Different viruses had different rates of infection under similar spinoculation conditions, and the difference was not related to the various sizes or weights of viral particles. In addition, the rate of viral infection is not the same when the same type of virus is used with the same centrifugal conditions but with different cell lines [15]. In a study spinoculation was used as a substitute for heparan sulfate for herpes simplex virus (HSV-1) infection of glycosaminoglycan-deficient cells. The heparan sulfate acts as a co-receptor for the HSV-1 virus to bind to the cell surface [16].

Based on the above-mentioned issues, it seems that spinoculation concentrates not only on viral particles but also causes physiological and biochemical changes at the cell surface, leading to an increase in the permissiveness of the target cell for viral infection. A recent study reported that the spinoculation facilitates HIV-1 infection of T cells by triggering cortical actin and cofilin activity [17]. In another study, the cortical actin was a significant barrier to T cell infection by HIV [18]. They showed that actin molecules polymerize to resist pressure during centrifugation, which activates the cofilin protein leading to the depolymerization of actin filaments. The dynamic between actin and caffeine is required for T cell infection by HIV-1, which is altered by proteins such as gp120 and Nef [17, 19]. In addition, spinoculation may induce chromatin remodeling and facilitate the entry of HIV-DNA into the genome of the infected cells.

On the other hand, the ability of the HIV-1 virus is limited to infect resting T cells [20], which may be related to the inability of the HIV-1 virus to transfer its DNA into the nucleus of an inactive cell, as well as its inability to find an open chromatin location [8].

**Fig. 4:**
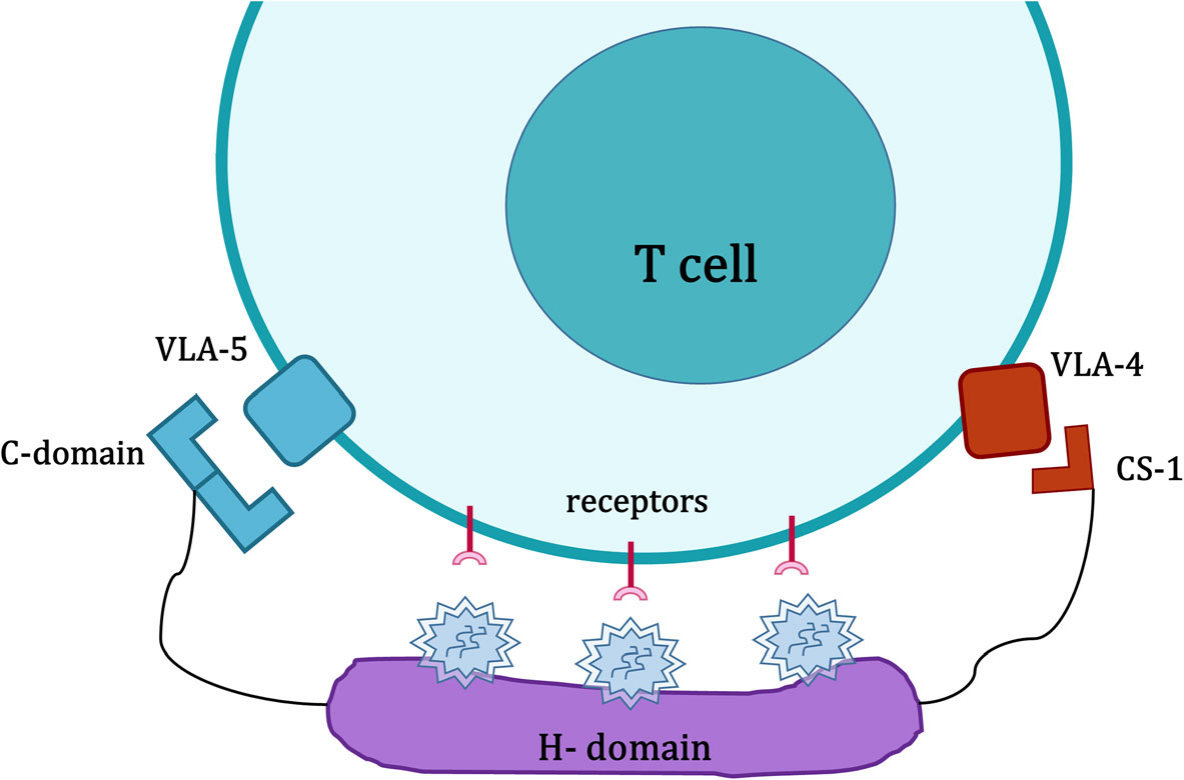
Retronectin increases the efficiency of cell-virus binding and infection rate

In this study, retronectin was used as a booster for the transduction of activated T cells. Retronectin is a fragment of recombinant human fibronectin with three domains. The C-domain and CS-1 are responsible for binding the peptide to the target cell, and the H-domain is responsible for binding to and trapping viral particles. Cell-binding domain receptors in the target cell are VLA-5 and VLA-4, respectively (Fig. 4). These receptors exist on the surface of lymphocytes. Therefore, the use of retronectin is effective in the transduction efficiency of these cells. The results are consistent with those of Rustanti et al., in which spinoculation increased the T cell transduction rate significantly. Retronectin association with spinoculation led to a 25% increase in T cell transduction rate compared to polybrene and spinoculation [18].

## Conclusion

Based on our results, the use of retronectin and spinoculation has a synergistic effect on the transduction rate of the lentiviral vector into human T cells, which may be effective in increasing the production of engineered T lymphocytes, leading to an increase in the success of CAR T cell-based therapies.

## Material and method

### Cell line packaging and production of viral particles

HEK-293 T (Iranian Biological Resources Center) cells were seeded to three 100 cm^2^ plates (2.5 × 10^6^ cells) and incubated overnight at 37 °C and 5% CO_2_. During the next day, transfer vector DNA (PLJM1-EGFP), together with pMDLg/pRRE packaging plasmid, pMD2.G envelop plasmid, and pRSV-Rev plasmid was co-transfected to HEK-293 T cells at a ratio 4:2:1:1 respectively. The transfection protocol was performed by three methods; lipofectamine2000™ Reagent (Invitrogen), polyethyleneimine (PEI) (Sigma), and calcium phosphate (CaPO4) methods. The transfection rates were evaluated by a fluorescent microscope.

### Absolute qPCR for viral titration

Cell supernatant containing viral particles was collected at 1-, 2-, and 3 days post-transfection and was centrifuged at 1500 rpm for 5 min at 4 °C. Then lentivirus-containing media were passed through a 0.45 μm pore filters (Millipore). Lentiviruses were concentrated by ultra-centrifugation for 120 min at 20000×g / 4 °C. The supernatant was removed and pellets resuspended in 200 μL of DMEM, taken aliquots, and stored at − 80 °C.

HEK-293 T cells were transduced by MUC1 CAR containing lentiviral vectors and evaluated by quantitative real-time PCR in order to determine the lentiviral particle titer. For this purpose, 5 × 10^4^ cells were plated in 24 well plates 24 h before transduction. Subsequently, the cell supernatant was removed and exposed to 50 μL concentered lentiviral particles in a final volume of 300 μL DMEM was supplemented with 5 μg/mL hexadimethrine bromide (polybrene, Sigma-Aldrich). Plates were incubated overnight at 5% CO_2_, 37 °C incubators. The next day, transduction medium was replaced with fresh complete DMEM. The cells were detached from plates and were followed by DNA extraction (DNA extraction kit, Roche) 72 h after transduction. Viral titers was assessed using detection of the puromycin resistance gene by qPCR as previously described [9];

### Preparation of human T cells

Peripheral blood samples were obtained from healthy donors in EDTA green top tubes after getting informed consent and in compliance with the ethical principles of Tehran University of Medical Sciences guidelines for human researches (approval code: IR.TUMS.VCR.REC.1395.538). Peripheral blood mononuclear cells (PBMC) were separated using ficoll-paque (Sigma) reagent. Survival cells were counted by trypan blue using the Neubauer chamber. Then, the activation of T lymphocytes was performed with anti-CD3/CD28 antibodies. PBMCs (10^6^ cells) were cultivated in complete medium (FBS 10%-RPMI 1640) and 100 IU/mL rIL-2. Then, cells mixed with Human T-Activator CD3/CD28 Dynabeads™ (Gibco) at a 1:1 bead-to-cell ratio. The beads were removed after 48-72 h, and CD3^+^cells were analyzed by flow cytometry.

### T lymphocytes transduction

Stimulated human primary T lymphocytes were transduced with five different methods for MUC1CAR lentiviral vectors.

#### Hexadimethrine bromide (polybrene)

For this method, 0.5 × 10^6^ activated T cells were incubated in 0.5 ml RPMI medium supplemented with 5% FBS, concentrated viral supernatant (MOI ≥ 10) and 8 μg/mL polybrene (Sigma) with gently mixing. Polybrene was removed during the next day, and 0.5 ml of complete medium, polybrene, 100 IU/mL of rIL-2, and concentrated viral supernatant were added to the T lymphocytes. After 48–72 h, cells were washed and prepared for analysis.

#### RetroNectin

The activated T cells (0.5 × 10^6^ cells) were incubated in 0.5 ml of complete RPMI medium and concentrated viral supernatant (MOI ≥ 10) in 24 well plates precoated with RetroNectin (10 μg/cm^2^) (Takara Bio) with periodical mixing. After overnight, the transduction medium was replaced with a fresh complement medium, concentrated viral supernatant, and rIL-2 (100 IU/mL). After 48–72 h, cells were washed and prepared for analysis.

#### Spinoculation method

For the transduction method, 0.5 × 10^6^ activated T cells were incubated in 0.5 ml RPMI medium was supplemented with 5% FBS and concentrated viral supernatant (MOI ≥ 10) in 24 well plates for 20 min. In the next step, the cells were centrifuged at 800×g/ 90 min/ 32 °C. In the next procedure, the cells were resuspended and transferred to the CO_2_ incubator. In addition, the fresh transduction medium supplemented with 100 IU/ml rIL-2 was replaced during the next day. After 48–72 h, cells were washed and prepared for analysis.

#### Spinoculation+RetroNectin

This method was the same procedure mentioned in the spinoculation method, but the cells were incubated in the precoated 24 well plates with RetroNectin (10 μg/cm2) (Takara Bio).

#### Spinoculation+Polybrene

The procedure was the same method mentioned in the spinoculation method except for the polybrene of transduction medium (8 μg/mL).

### Flowcytometry

For surface staining, 2–5 × 10^5^ transduced or untransduced T cells were rinsed in PBS supplemented with 10% FBS and 0.05% sodium azide), and subsequently stained with either APC-conjugated anti-CD3 (BD Pharmingen) for evaluating CD3^+^ T cell population or primary rabbit anti-VHH and secondary FITC-conjugated anti-rabbit IgG (Abcam) for evaluating the transduction rate. After 30 min on ice, samples were assessed by BD FACSCanto II equipment, and the data were analyzed by FlowJo software (v10).

### Cytotoxicity

Transduced T cells were analyzed by MTT assay after 72 h of transduction to determine the cytotoxicity of transduction methods. For this purpose, the cells were centrifuged in the 96 plates at 1000×g for 5 min, and MTT solution was added to all wells after aspiration. Then, the cells were incubated for 3 h. DMSO was added to wells after the purple formazan formation, and the absorbance was detected at 540 nm wavelength by a microplate reader.

## Supplementary Information

**Additional file 1.**

## Data Availability

The viral titration results can be found in the supplementary information. The datasets used and/or analyzed during the current study are available from the corresponding author on reasonable request.

## Abbreviations

ALL: Acute lymphoblastic leukemia
BCR: B cell receptor
CAR: Chimeric antigen receptor
MOI: Multiplicity of infection
MUC1: Mucin-1
PBMC: Peripheral blood mononuclear cell
PEI: Polyethyleneimine
ScFv: Single-chain variable fragment

## References

[1] Nie Y, Lu W, Chen D, Tu H, Guo Z, Zhou X, Li M, Tu S, Li Y (2020). Mechanisms underlying CD19-positive ALL relapse after anti-CD19 CAR T cell therapy and associated strategies. Biomark Res.

[2] Han D, Xu Z, Zhuang Y, Ye Z, Qian Q (2021). Current progress in CAR-T cell therapy for hematological malignancies. J Cancer.

[3] Taylor-Papadimitriou J, Burchell JM, Graham R, Beatson R (2018). Latest developments in MUC1 immunotherapy. Biochem Soc Trans.

[4] Bajgain P, Tawinwung S, D’Elia L, Sukumaran S, Watanabe N, Hoyos V, Lulla P, Brenner MK, Leen AM, Vera JF (2018). CAR T cell therapy for breast cancer: harnessing the tumor milieu to drive T cell activation. J Immunother Cancer.

[5] Posey AD, Schwab RD, Boesteanu AC, Steentoft C, Mandel U, Engels B, Stone JD, Madsen TD, Schreiber K, Haines KM, Cogdill AP, Chen TJ, Song D, Scholler J, Kranz DM, Feldman MD, Young R, Keith B, Schreiber H, Clausen H, Johnson LA, June CH (2016). Engineered CAR T cells targeting the cancer-associated Tn-glycoform of the membrane mucin MUC1 control adenocarcinoma. Immunity.

[6] Fleischer LC, Spencer HT, Raikar SS (2019). Targeting T cell malignancies using CAR-based immunotherapy: challenges and potential solutions. J Hematol Oncol.

[7] Poorebrahim M, Sadeghi S, Fakhr E, Abazari MF, Poortahmasebi V, Kheirollahi A, Askari H, Rajabzadeh A, Rastegarpanah M, Linē A, Cid-Arregui A (2019). Production of CAR T-cells by GMP-grade lentiviral vectors: latest advances and future prospects. Crit Rev Clin Lab Sci.

[8] Fichter C, Aggarwal A, Wong AKH, McAllery S, Mathivanan V, Hao B, MacRae H, Churchill MJ, Gorry PR, Roche M, Gray LR, Turville S (2021). Modular lentiviral vectors for highly efficient transgene expression in resting immune cells. Viruses..

[9] Rajabzadeh A, Rahbarizadeh F, Ahmadvand D, Salmani MK, Hamidieh AA (2021). A VHH-based anti-MUC1 chimeric antigen receptor for specific retargeting of human primary T cells to MUC1-positive cancer cells. Cell J.

[10] Tang Y, Garson K, Li L, Vanderhyden BC (2015). Optimization of lentiviral vector production using polyethylenimine-mediated transfection. Oncol Lett.

[11] Sou SN, Polizzi KM, Kontoravdi C (2013). Evaluation of transfection methods for transient gene expression in Chinese hamster ovary cells. Adv Biosci Biotechnol.

[12] Pirona AC, Oktriani R, Boettcher M, Hoheisel JD (2020). Process for an efficient lentiviral cell transduction. Biol Methods Protoc.

[13] Maginnis MS (2018). Virus–receptor interactions: the key to cellular invasion. J Mol Biol.

[14] Ghaleh HEG, Bolandian M, Dorostkar R, Jafari A, Pour MF (2020). Concise review on optimized methods in production and transduction of lentiviral vectors in order to facilitate immunotherapy and gene therapy. Biomed Pharmacother.

[15] Yan R, Zhang Y, Cai D, Liu Y, Cuconati A, Guo H (2015). Spinoculation enhances HBV infection in NTCP-reconstituted hepatocytes. PLoS One.

[16] de Mello CP, Bloom DC, Paixão IC (2016). Herpes simplex virus type-1: replication, latency, reactivation and its antiviral targets. Antivir Ther.

[17] Ospina Stella A, Turville S (2018). All-round manipulation of the actin cytoskeleton by HIV. Viruses.

[18] Rustanti L, Jin H, Li D, Lor M, Sivakumaran H, Harrich D (2018). Differential effects of strategies to improve the transduction efficiency of lentiviral vector that conveys an anti-HIV protein, Nullbasic, in human T cells. Virol Sin.

[19] Lamas-Murua M, Stolp B, Kaw S, Thoma J, Tsopoulidis N, Trautz B, Ambiel I, Reif T, Arora S, Imle A, Tibroni N, Wu J, Cui G, Stein JV, Tanaka M, Lyck R, Fackler OT (2018). HIV-1 Nef disrupts CD4+ T lymphocyte polarity, extravasation, and homing to lymph nodes via its Nef-associated kinase complex interface. J Immunol.

[20] Pan X, Baldauf H-M, Keppler OT, Fackler OT (2013). Restrictions to HIV-1 replication in resting CD4+ T lymphocytes. Cell Res.

