# Supplementary material for: Spinoculation and retronectin highly enhance the gene transduction efficiency of Mucin-1-specific chimeric antigen receptor (CAR) in human primary T cells
Source: BMC Mol Cell Biol. 2021 Nov 23;22:57. doi: 10.1186/s12860-021-00397-z (PMC8609792; doi:10.1186/s12860-021-00397-z)
Supplement: Supplementary file 1 — Additional file 1. [file 12860_2021_397_MOESM1_ESM.docx]

**
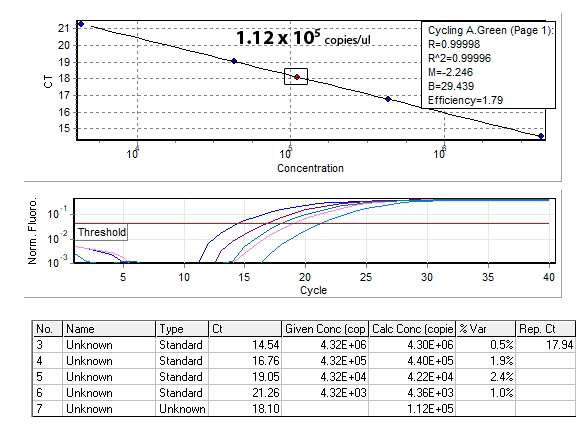
**

**S1.** Copy numbers of integrated lentiviral vectors were measured by the standard curve of real time PCR of puromycin resistant gene (Rotor-Gene 6000 Series Software 1.7). The titer of recombinant anti-MUC1 CAR lentiviral vector in transforming unit per mL was calculated 1.12 x 10^8^ TU/mL from real time PCR results. The image represents standard curve of quantification of virus copy numbers in 10-fold-dilutions (4.32 X 10^6^-4.32 X 10^3^ viral copies/ul). The results indicates CAR recombinant lentiviral titer was 1.12 X 105 viral particle/ul. DNA copy number of standards was calculated by this formula: **number of copies = (amount (ng) * 6.022x1023) / (length (bp) * 1x10^9^ * 650)**


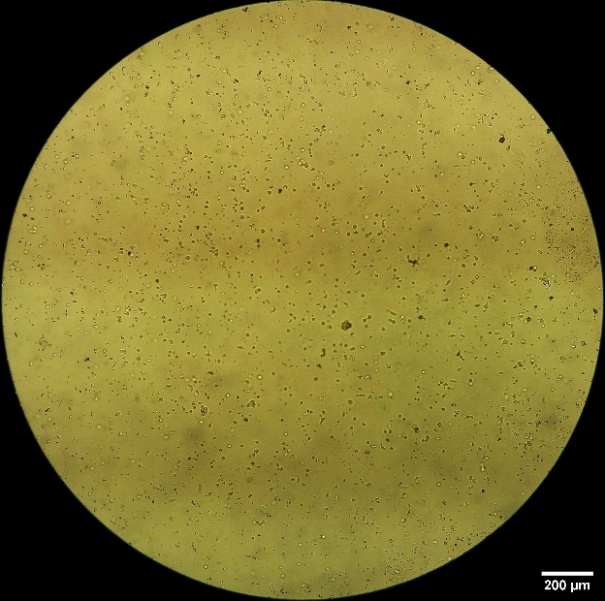

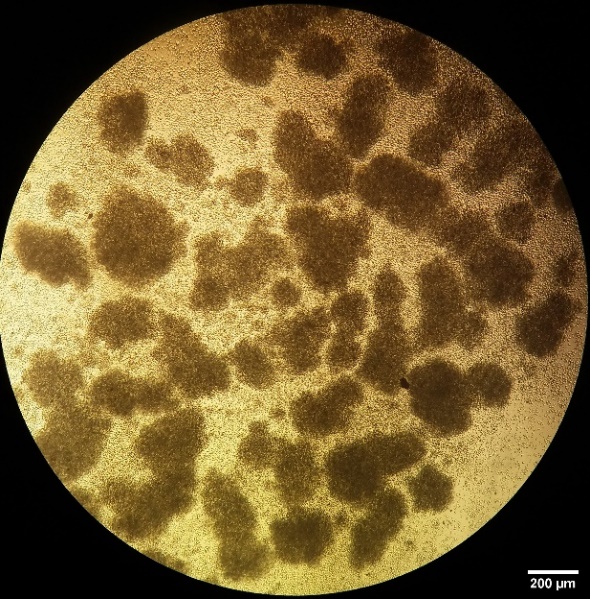


**S2.** Stimulation and enrichment of CD3^+^ T cells 48h after treatment by CD3/CD28 Dynabeads^TM^ T-activator
